# Zika Travel Policies May Reduce Women's Leadership in Global Health

**DOI:** 10.9745/GHSP-D-16-00282

**Published:** 2016-12-23

**Authors:** Emma Richardson

**Affiliations:** a McMaster University, Ontario, Canada.

On February 1, 2016, the World Health Organization (WHO) declared Zika a “Public Health Emergency of International Concern,” based on growing evidence that women who have theZika virus during pregnancy are at increased risk of having their children born with microcephaly.^1^ On November 20, 2016, while this letter was in press, WHO declared that Zika was no longer a Public Health Emergency of International Concern because the link between Zika and microcephaly has been found to be robust and in need of a longer-term global strategy.^2^^,^^3^ To stem the spread of Zika, travel-related policies have been issued by federal public health agencies and are still in place, advising in particular pregnant women or women trying to become pregnant not to travel to areas with ongoing Zika virus outbreaks.^4^ These policies may have the unintended result of decreasing women's input on the planning, implementation, and evaluation of global health projects. This is important to the field as a whole, because gender-balanced teams are crucial for implementing effective global health programs and projects. As a woman global health scholar and practitioner, I reflect on potential negative impacts of these Zika travel policies and recommend actions.

Gender balance is important to effective global health programs.

## ZIKA TRAVEL POLICY AND GLOBAL HEALTH

In the United States, the Centers for Disease Control and Prevention (CDC) announced, on January 15, 2016, a travel alert for 14 Zika-affected countries in Latin America, the most economically unequal region in the world^5^ with considerable and persistent global health challenges. This region continues to be a high priority for health donors such as the U.S. Agency for International Development.^6^ The travel policy recommends special precautions for women who are pregnant or trying to become pregnant, including considering postponing travel to these destinations.^7^ The Zika travel policy is unusual in its sex specificity: It recommends that women, and not men, should potentially avoid travel to Latin America. In fact, men can also become infected with Zika, which is sexually transmissible to their partners, but this scenario has not been addressed in the travel policies directly.^6^

The Zika travel policy is unusual in its sex specificity.

## GENDER-SPECIFIC IMPACTS OF THE U.S. ZIKA TRAVEL POLICY

1. **Women's leadership and input in global health programs may be reduced.** The status of “trying to become pregnant” is complex and may last for years. Many female global health profession-als of reproductive age, if they heed the advice of the CDC, would avoid work-related travel to Central and South America. This could mean that for the current cohort of global health projects, women's input would be significantly reduced. Lack of gender balance is known to hamper effective implementation of global health initiatives.^8^
2. **Women may be hesitant to express their concern about Zika.** Citing Zika travel policies as a reason to avoid work-related international travel may signal to employers a woman's intention to have children. Many women worry about revealing their plans to have children to their employers. Women are aware that revealing an intention to become pregnant might be detrimental to their career advancement. For example, employers may prefer to hire and promote those they suspect less likely to have children, to avoid having to cover maternity leaves. While the practice is technically illegal in the United States, women are often discriminated against in hiring, such as by being asked in job interviews if they plan to have children.^9^

Women are aware that revealing an intention to become pregnant might be detrimental to their career advancement.

## RECOMMENDATIONS FOR GLOBAL HEALTH FUNDERS AND IMPLEMENTING ORGANIZATIONS

1. **Monitor and report on whether women's participation in global health programs and policies is decreasing.** Compare measures of participation before and after Zika travel policies were in place.
2. **Mitigate the impact.** Seek more flexible ways to involve women in global health projects. For example, I acted as gender consultant for a maternal health initiative in Guatemala by combining a shorter in-country trip with later teleconferencing for interviews with maternal health project staff based in Northern Guatemala.
3. **Consider increasing the participation of local as opposed to expatriate women.** The reality is that most global health projects are funded and managed by international institutions headquartered in the North that employ many expatriates. If instead local professional women were brought to the fore, this could be a silver lining. Involving more local women would improve adaptation of the project to the local context and mitigate the overall loss of women's perspectives resulting from the Zika travel policies.

Involving more local professional women in global health projects could be a silver lining.

## RECOMMENDATIONS FOR INDIVIDUAL WOMEN WHO WORK IN GLOBAL HEALTH

1. **Know you are not alone.** For women of reproductive age who are involved in global health, navigating adherence to Zika travel policies is complex. I hope this letter will stir reflection and push these issues from private dilemmas to public debate.
2. **Suggest to global health employers alternative ways for women to be involved.** If your intention to become pregnant means you cannot risk travel to Zika-endemic areas, suggest to your employer alternatives such as teleconferencing or partnering with a local counterpart who is a woman.

Zika travel policies may have the unintended consequence of reducing women's participation in global health programs. Addressing gender equity in global health projects is complex. Recognizing and dealing with gender imbalances in global health leadership is perhaps more subtle, but no less important.
